# Gender-Based and Bilateral Variations in the Quadriceps Angle and Femoral Bicondylar Distance Among Healthy North Indian Adults: A Cross-Sectional Study

**DOI:** 10.7759/cureus.113310

**Published:** 2026-07-24

**Authors:** Rahul Sharma, Gitanjali Khorwal, Kirandeep K Aulakh, Harpreet Kaur Mohel

**Affiliations:** 1 Anatomy, Maharishi Markandeshwar Institute of Medical Sciences and Research, Mullana, IND; 2 Anatomy, All India Institute of Medical Sciences Rishikesh, Rishikesh, IND; 3 Anatomy, Maharishi Markandeshwar University, Mullana, IND

**Keywords:** femoral bicondylar distance, gender difference, knee alignment, knee biomechanics, quadriceps angle (q-angle)

## Abstract

Introduction: The quadriceps angle (Q-angle) and bicondylar distance (ICD) are physical assessments used for knee alignment. Both parameters provide valuable insights into gender-specific and bilateral variations in knee biomechanics that may influence injury risk, functional performance, and degenerative changes throughout adulthood.

Materials and methods: This cross-sectional, observational study was conducted to evaluate gender-based variations in the Q-angle and ICD of the femur, comprising 600 participants (300 male participants and 300 female participants), aged 20-50 years, of North Indian origin. The Q-angle was measured using a universal 360° manual goniometer, and the ICD of the femur was measured using a digital Vernier caliper in accordance with standard anthropometric measurement procedures.

Results: The average age of male participants was 35.4 ± 9.0 years, while that of female participants was 35.5 ± 8.6 years in the study population. Male participants had a slightly higher mean height (164.8 ± 8.2 cm) and mean body weight (66.6 ± 8.5 kg). Female participants demonstrated significantly higher Q-angle values than male participants (16.53± 5.22 vs 13.85 ± 5.32, p < 0.001), whereas the ICD does not show a significant difference between genders (p = 0.083). Within gender groups, significant right-left differences were observed for the Q-angle.

Conclusion: The study provides valuable normative data on the Q-angle and ICD in the adult North Indian population. Female participants showed significantly higher Q-angle values than male participants, while the ICD showed no gender related difference, suggesting that Q-angle and ICD parameters represent distinct aspects of knee alignment and independently contribute to knee biomechanics.

## Introduction

The quadriceps angle (Q-angle) and bicondylar distance (ICD) are critical biomechanical parameters that reflect lower limb alignment and functional loading of the knee joint [1]. Brattstrom (1964) represents the Q-angle as a resultant line of pull of the quadriceps muscle compared to the patellar tendon and is commonly used to assess patellofemoral joint mechanics. An increase in value mainly results in anterior knee pain, patellofemoral syndrome, and knee instability [2]. The ICD, measured between the medial and lateral femoral condyles of knees, serves as a secondary indicator of frontal plane knee alignment and genu varum-valgum characteristics [3].

The lower limb alignment differs with gender, as adult women show an increased Q angle compared to adult men. The difference occurs due to an increased pelvic width, an increase in femoral anteversion, valgus at the tibiofemoral joint, and laxation of ligaments [4,5]. Changes in the anatomical and postural alignment can result in alteration of force distribution along the patellofemoral joint, potentially causing an increase in lateral patellar tracking and increasing the stress load on the lateral femoral condyle. Compared to that, men show a smaller Q-angle and a larger ICD, showing relatively neutral and varus knee alignment [6]. Such examination of the gender-specific Q-angle and ICD together is more informative for understanding alignment [5].

Beyond gender differences, the Q-angle and ICD show bilateral similarity, although a smaller left vs. right difference can be seen or arise due to limb dominance, habitual loading patterns, or occupational need and past limb injury [7]. In existing literature, it has been seen that there is a bilateral Q-angle difference in both asymptomatic and symptomatic populations, suggesting that there is a greater mechanical stress on the predisposed limb during different functional activities such as walking, stair climbing, or prolonged standing over a dominant limb [8-10].

Anthropometric factors such as age, weight, height, and body mass index have also been measured in relation to the Q-angle and ICD and show a positive correlation between weight and age with the Q-angle, particularly in women [11,12]. An increase in weight results in increased stress on the lower limb, which may result in lower limb alignment deviations, thereby amplifying biomechanical stress on the knee joint [10]. In the adult population, this parameter becomes more important as this group falls in transition periods wherein an increase in mechanical load may induce early degenerative or overuse conditions [13].

Despite numerous studies done on the Q-angle, comparatively fewer studies have been conducted for the ICD in the adult population, particularly with respect to gender and bilateral baseline comparison. The ICD is a simple, cost-effective physical clinical measurement that provides linear information about the knee alignment in the frontal plane, in contrast to angular assessment [1]. When measured together, the Q-angle and ICD are said to provide a more comprehensive representation of lower limb alignment, measuring both angular and spatial characteristics of the knee joint. The present study aims to evaluate gender-based and bilateral variation in the Q-angle and ICD and to determine the correlation between these parameters among healthy North Indian adults aged 20-50 years to help establish the normative value and clarify the extent of right-to-left variations with age, height, and body weight. Such normative values are required for ergonomic assessment, injury prevention, and early identification of individuals at risk of developing patellofemoral disorder and degenerative knee conditions.

## Materials and methods

This cross-sectional, observational study was performed to evaluate gender-based variations in the Q-angle and ICD of the femur in an adult Indian population, including 600 participants, 300 male participants and 300 female participants, aged 20-50 years, of North Indian origin. This study was done in the Department of Anatomy, MMIMSR, Ambala, Haryana, following approval from the Institutional Ethics Committee (IEC NO 3511).

This study included adult participants aged 20-50 years without lower-limb deformities. Informed consent was obtained before the study in both vernacular and English languages. The study excluded participants with a history of trauma, surgery in the lower limbs, musculoskeletal or neurological disorders, or any gait abnormalities.

The height of participants was measured using a standard stadiometer (PrimeSurgicals height measuring scale; PrimeSurgicals, New Delhi, India). Participants stood barefoot in an erect posture with heels slightly apart, back straight, and head in the Frankfurt plane. The measurement was recorded to the nearest 0.1 cm. 

Body weight was measured using a calibrated digital weighing scale (Thermomate Deluxe product CAMRY, digital weighing scale, CAMRY Electronic Co., Ltd., Zhongshan, Guangdong, China), with participants wearing light clothing and no footwear. Values were recorded to the nearest 0.1 kg.

**Figure 1:**
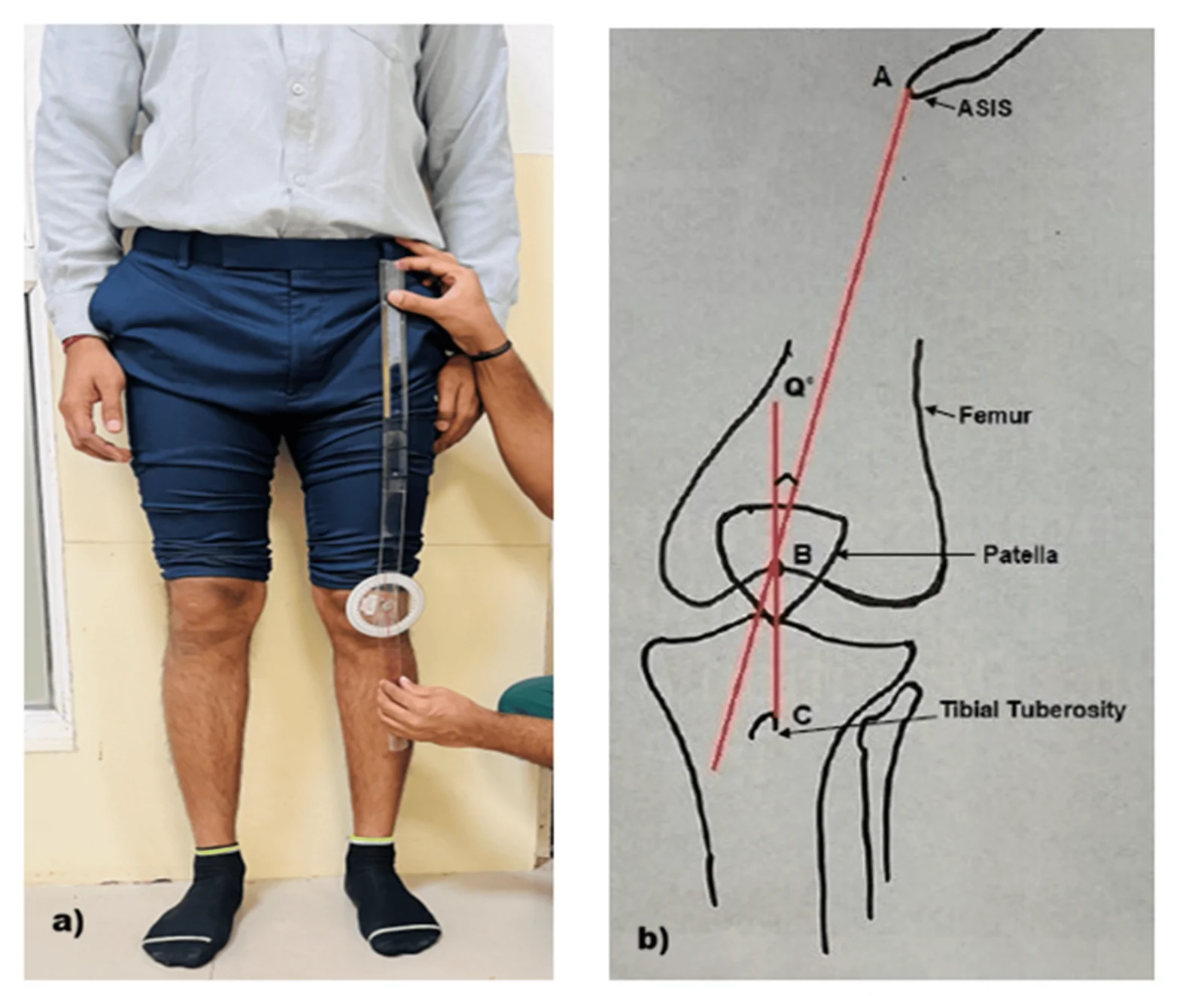
a) Measurement of the Q-angle in standing position by a universal 360° goniometer, and b) line diagram for measuring the Q-angle (Q°), showing the following reference points: (A) Anterior superior iliac spine (ASIS); (B) midpoint of the patella; (C) tibial tuberosity. (b) Image credit: Rahul Sharma (first author), and software used for marking the illustration was Paint software, 11.2603.251.0, Microsoft 2026 (Microsoft Corporation, Redmond, USA).

The Q-angle was measured using a universal manual goniometer (goniometer degree protector angle scale, LISAMED, Coimbatore, India), whose intraclass correlation coefficient consistently exceeded 0.90 [14], following standardized anatomical procedures. The participant was positioned in a standing, weight-bearing posture, with hips and knees fully extended, quadriceps relaxed, and feet placed in a neutral position. Anatomical landmarks were identified and marked as (i) anterior superior iliac spine (ASIS), (ii) center of the patella, defined as the point of intersection of the longest vertical line and transverse diameter of the patella, and (iii) the highest bony prominence of the tibial tuberosity. The axis of the goniometer was placed over the center of the patella. One limb of the goniometer was aligned with the line joining the ASIS to the patella. The other was aligned with the line joining the tibial tuberosity to the patella. The angle formed between these two lines was recorded as the Q-angle (Q°), as shown in Figures 1a, 1b.

**Figure 2:**
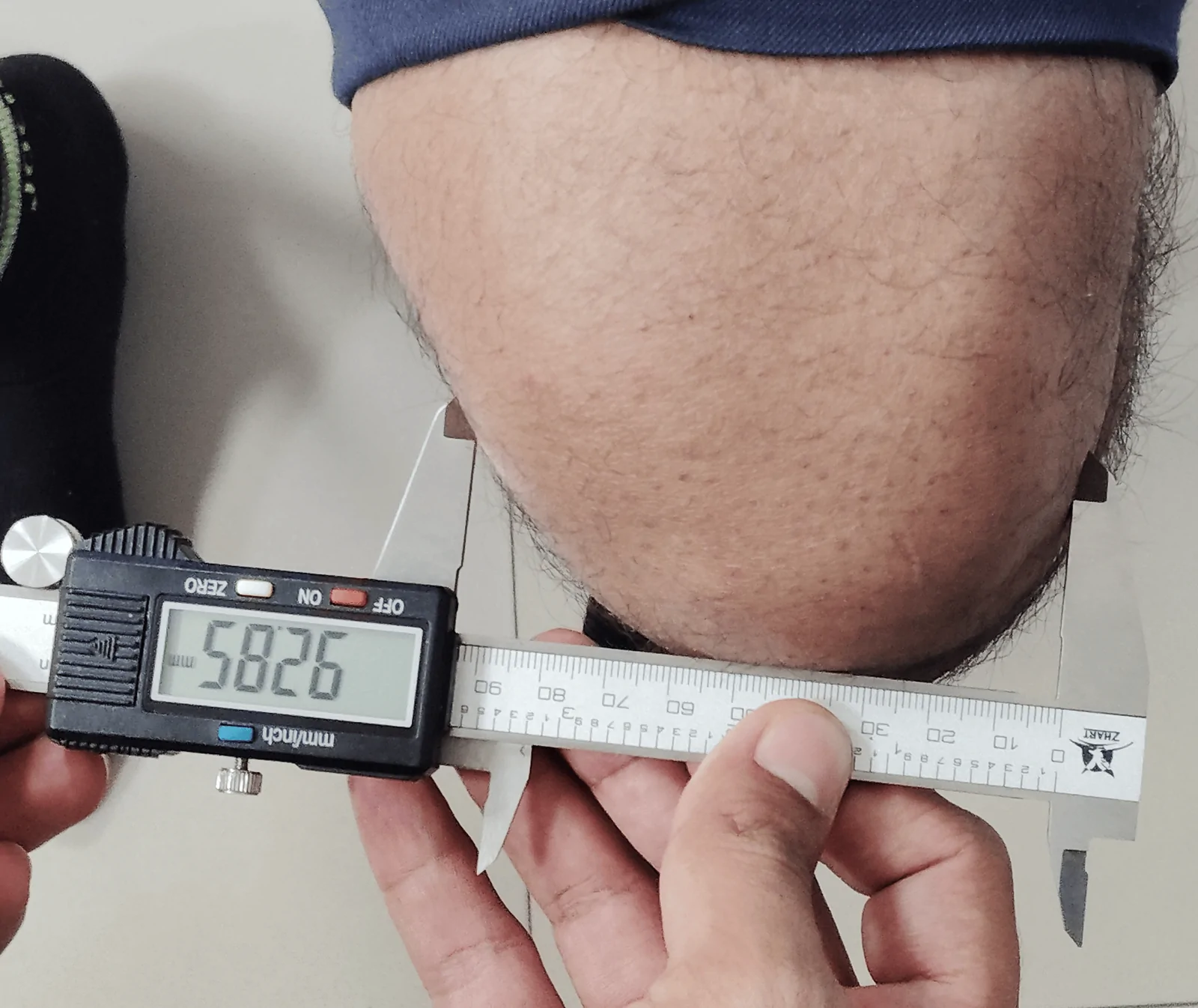
Measurement of the bicondylar distance (ICD) by using a digital vernier caliper.

The ICD of the femur was measured using a digital Vernier caliper scaled from 0 cm to 20 cm and with a marginal error of ±1mm (Zhart Vernier Caliper Digital 150mm/6 inch Video Display measurement tool; Zhart, Shenzhen, Guangdong, China) in accordance with standard anthropometric procedures. The participant was seated with the knees flexed to 90° and feet flat, facing forward. The medial and lateral femoral condyles were carefully palpated and identified. The immovable jaw of the caliper was positioned on the lateral condyle, while the moving jaw was positioned on the medial condyle. The distance between the two condyles was then recorded in millimeters (mm), converted into centimeters (cm), as shown in Figure 2. A single trained investigator performed all measurements to ensure consistency and reduce inter-observer variability. Three measurements were recorded for each side, and the average value was noted.

Data were recorded and incorporated into Microsoft Excel and analyzed using IBM SPSS Statistics for Windows, Version 27 (Released 2019; IBM Corp., Armonk, New York, United States).

Statistical analysis

Descriptive statistics were used to calculate the mean ± standard deviation (SD) for age, height, and weight. Normality of the sample was evaluated using the Shapiro-Wilk W-statistic for male and female participants (p>0.05), which revealed that the ICD was normally distributed, while the Q-angle deviated from normality.

Non-parametric tests were employed to draw statistical inferences regarding the Q-angle, as the data were not normally distributed. The Mann-Whitney U test was conducted to compare the male and female groups, and the Wilcoxon signed-rank test was applied to determine the difference between paired variables on the right and left sides in male participants and female participants.

Parametric tests were applied to draw statistical inferences regarding the ICD, as data were normally distributed. A paired-sample t-test was used to assess the difference between the right and left side variables in male participants and female participants. An independent-samples t-test was used for comparison of normally distributed male and female groups. Spearman's correlation coefficient (r) was used to evaluate the relationships between the Q-angle and ICD for all participants. A significance level of p < 0.05 was adopted.

## Results

The demographic characteristics of the study population were comparable between male participants and female participants. The mean age of male participants was 35.4 ± 9.0 years, and that of female participants was 35.5 ± 8.6 years, indicating a similar age distribution across both groups. Male participants had a slightly higher mean height (164.8 ± 8.2 cm) compared to female participants (163.7 ± 9.2 cm). Similarly, the mean body weight was higher in male participants (66.6 ± 8.5 kg) than in female participants (63.3 ± 9.1 kg), as shown in Table 1. 

**Table 1:** Mean ± standard deviation (SD), (p < 0.05), and n denotes the number of participants in each group (male = 300, female = 300). Age is expressed in years, height in centimeters (cm), and weight in kilograms (kg).

Variable	Male (Mean ± SD) n=300	Female (Mean ± SD) n=300
Age (years)	35.4 ± 9.0	35.5 ± 8.6
Height (cm)	164.8 ± 8.2	163.7 ± 9.2
Weight (kg)	66.6 ± 8.5	63.3 ± 9.1

The comparison of the Q-angle between male participants and female participants revealed a statistically significant difference, with female participants demonstrating higher Q-angle values (16.53 ± 5.22) compared to male participants (13.85 ± 5.32°). The mean difference was -2.96° (95% CI: -3.28 to -2.09), which was highly significant (p < 0.001), and the strength of association (Point-Biserial correlation) was 0.25 (medium effect size). This indicates a clear gender-based variation in the Q-angle.

In contrast, the ICD does not show a statistically significant difference between male participants (9.39 ± 1.33 cm) and female participants (9.26 ± 1.36 cm). While male participants showed slightly higher values, the mean difference of 0.27 cm (95% CI: -0.02 to 0.28) was not statistically significant (p = 0.083), and the strength of association (Point-Biserial correlation) was 0.05 (little/no association), as shown in Table 2.

**Table 2:** Comparison of the Q-angle and bicondylar distance (ICD) in male and female genders. To make a comparison, the Q-angle in male participants and female participants was tested using the Wilcoxon-Mann-Whitney U test, and for the ICD, parametric tests (t-test) were used to make group comparisons. Q-angle: Quadriceps angle; ICD: bicondylar distance

Variable	Parameter	Total	Gender		Difference (95% CI)	Significance	Test Statistic
			Male	Female			
Q-Angle (°)	Mean ± SD	15.19 ± 2.52	13.85 ± 5.32	16.53 ± 5.22	-2.69 (-3.28 to -2.09)	p = <0.001	W=65298.000
ICD (cm)	Mean ± SD	9.32 ± 1.33	9.39 ± 1.33	9.26 ± 1.36	0.13 (-0.02 to 0.28)	p = 0.083	t=1.736

In male participants, the mean Q-angle was slightly higher on the right side (14.48 ± 8.21°) compared to the left (13.92 ± 8.34°), and the difference was statistically significant (p = 0.029). Similarly, in female participants, the right side (16.52 ± 8.41°) showed marginally higher values than the left (16.02 ± 8.66°), and the difference was significant (p = 0.034). This shows that the Q-angle demonstrates significant bilateral asymmetry in both male and female genders. Numerical differences show stance dominance, representing that the pull of quadriceps muscles affects the knee alignment on both sides. 

The ICD, among male participants, the ICD was 9.27 ± 1.36 cm (right) and 9.25 ± 1.34 cm (left), with no significant difference (p = 0.970). Among female participants, ICD values were 9.27 ± 1.36 cm (right) and 9.32 ± 1.28 cm (left), also showing no significant difference (p = 0.362), as shown in Table 3. This shows that the ICD remains highly consistent between sides in both genders and indicates symmetrical condylar alignment and load distribution.

**Table 3:** Bilateral comparison of the Q-angle and bicondylar distance (ICD) in male and female participants. For the Q-angle, the Wilcoxon signed-rank test was performed due to non-normally distribution of data, with an effect size for male participants (r ≈0.126) and for female participants (r ≈0.122) (shows a small effect size), and the paired t-test was performed for the ICD as data was normally distributed with the effect size Cohen's d of 0.002 for male participants and 0.051 for female participants with degree of freedom (df 299). A p-value < 0.05 was considered statistically significant. Q-angle: Quadriceps angle; ICD: bicondylar distance

Variable	Right	Left	p-value	Test Statistic	Significant	Test
Q-Angle (°) (Male)	14.48 ± 8.21	13.92 ± 8.34	0.029	z= -2.18	Significant	Wilcoxon signed-rank test
Q-Angle (°) (Female)	16.52 ± 8.41	16.02 ± 8.66	0.034	z= -2.12	Significant	
ICD (Male)	9.27 ± 1.36	9.25 ± 1.34	0.970	t= 0.04	Not Significant	Paired t-test
ICD (Female)	9.36 ± 1.30	9.32 ± 1.28	0.379	t= -0.88	Not Significant	

**Figure 3:**
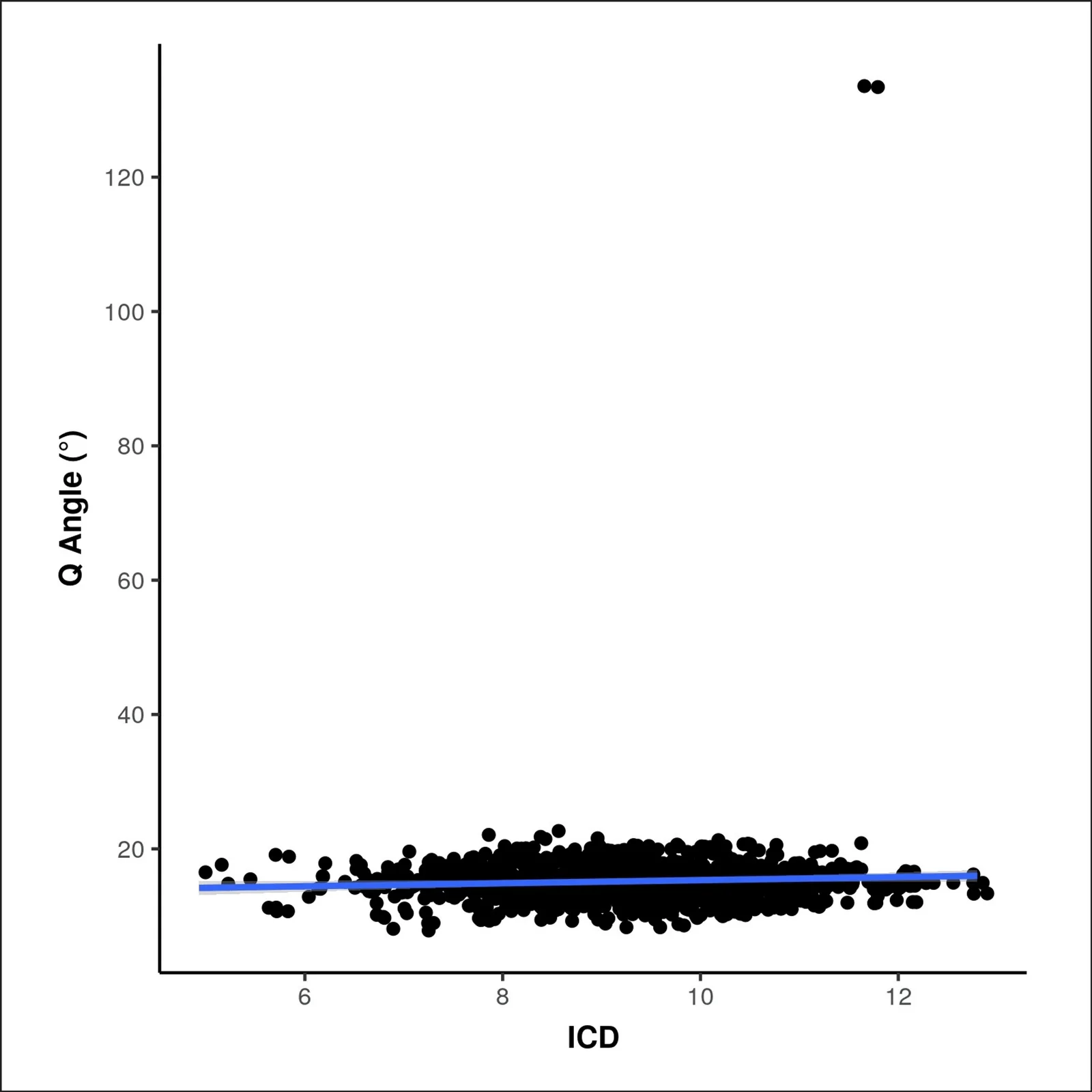
The scatterplot depicts correlation between ICD and Q-angle (°). Individual points represent individual cases. The blue trendline represents the general trend of correlation between the two variables. Q-angle: Quadriceps angle; ICD: bicondylar distance

Spearman’s correlation analysis demonstrated a very weak negative correlation between the Q-angle and ICD (ρ = -0.04) in overall male and female participants. However, this relationship was not statistically significant (p = 0.223). The 95% confidence interval (-0.09 to 0.02) includes zero, further confirming the absence of a meaningful association between the two. These findings indicate that changes in the ICD do not significantly influence the Q-angle in the studied population, as shown in Table 4 and Figure 3. 

**Table 4:** Spearman correlation analysis for the association between the Q-angle and ICD. Q-angle: Quadriceps angle; ICD: bicondylar distance

Correlation	Spearman Correlation Coefficient	p-Value
Q-Angle (°) vs ICD	-0.04	0.223

**Figure 4:**
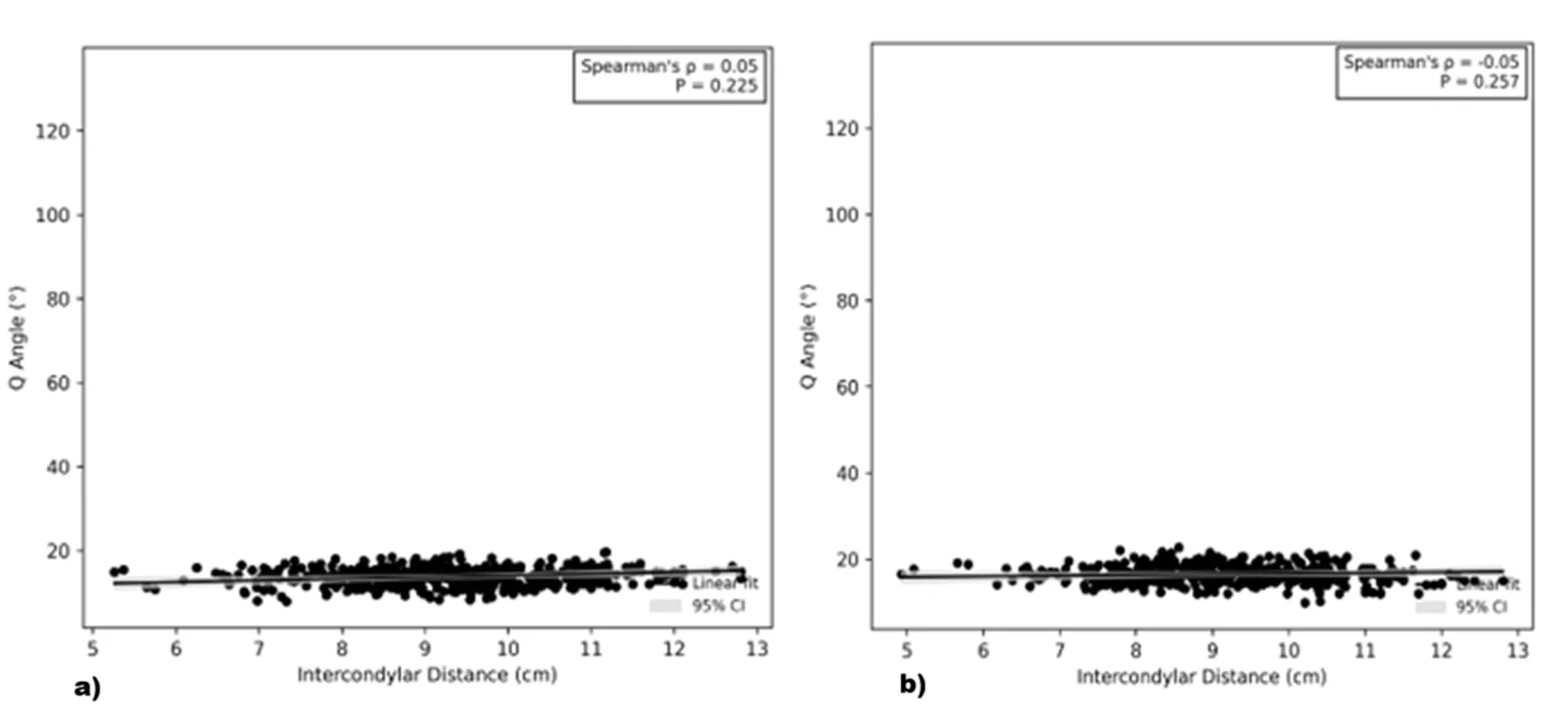
a) Scatter plot illustrating the relationship between quadriceps (Q) angle and bicondylar distance (ICD) in male participants (n = 300). b) Scatter plot illustrating the relationship between the quadriceps (Q) angle and ICD in female participants (n = 300). Each black dot represents one participant. The solid line represents the fitted linear regression line, while the shaded grey region denotes the 95% confidence interval of the regression estimate.

In male participants, Spearman correlation analysis demonstrated a very weak positive correlation between the ICD and Q-angle (ρ = 0.05), which was not statistically significant (p = 0.225). Similarly, female participants showed a very weak negative correlation between the Q-angle and ICD (ρ = -0.05), which is not statistically significant, as shown in Table 5 and Figures 4a, 4b. This indicates that the ICD was not a meaningful determinant of Q angle in the studied population. 

**Table 5:** Association between the Q-angle (°) and ICD in male and female genders. Q-angle: Quadriceps angle; ICD: bicondylar distance

Correlation	Spearman Correlation Coefficient (ρ)	p-Value
Q-Angle (°) vs ICD in male participants	0.05	0.225
Q-Angle (°) vs ICD in female participants	-0.05	0.257

## Discussion

The present study was done to evaluate gender-based bilateral differences in the Q-angle and ICD in an adult North Indian population. The findings of this study show that the Q-angle is statistically significant between male participants and female participants, with female participants demonstrating higher Q-angle values (16.53 ± 5.22°) compared to male participants (13.85 ± 5.32°). The ICD in male participants is 9.39 ± 1.33 cm and in female participants, it is 9.26 ± 1.36 cm. Though male participants exhibited slightly higher values than female participants, the difference between the two genders was not statistically significant.

A key finding of this study was the significantly higher Q-angle observed in female participants compared to male participants. This observation is consistent with previous studies, which have attributed this difference primarily to anatomical and biomechanical factors such as increased pelvic width, greater femoral anteversion, and lateral positioning of the tibial tuberosity in women [15,16]. Shantanu et al. reported that women consistently exhibit higher Q-angle values than men, which may increase the chance of developing patellofemoral disorders [15]. Similarly, Verma et al. found statistically significant gender differences, emphasizing the role of anatomical alignment in influencing Q-angle [16]. An increased Q-angle has been associated with lateral patellar tracking, patellofemoral pain syndrome, and a higher risk of ACL injuries [17,18]. These findings support biomechanical theory that altered alignment in females increases stress across the patellofemoral joint. Bhaskaran et al. (2016) further demonstrated that variations in Q-angle influence patellar stability and joint loading patterns [17].

In the present study, bilateral comparison revealed minimal differences between the right and left sides in both the Q-angle and ICD; however, the difference observed in the Q-angle was statistically significant. These findings were consistent with a study done by Kumar et al. (2023), suggesting that while bilateral asymmetry may exist, it is often small and may not be clinically significant and that slight differences between limbs may arise due to limb dominance or habitual activity patterns rather than pathological conditions [13]. 

ICD, on the other hand, did not show significant variation between males and females in this study. This suggests that ICD, as a linear measurement, may be less influenced by gender related anatomical differences compared to angular measurements like Q-angle. Similar findings have been reported in previous studies conducted by Omololu et al. (2003), where ICD remained relatively stable across different populations [19]. In contrast to the present study, Chauhan et al. (2017) performed a study on condylar distance on 100 dry femora. The condylar width of the femur was 73.11±6.14 mm on the right side and 72.16±6.58 mm on the left side. The study found that the condylar distance on the right limb was more often greater than that on the left side. Similarly, Kumar et al. (2023) show a positive correlation between the Q-angle and ICD in the 500-adult population (p-value = 0.0001) [13].

The lack of significant correlation between Q-angle and ICD suggests that these parameters should be assessed independently during clinical evaluation. A single parameter may not provide a complete understanding of knee alignment and biomechanics. This study also provides valuable normative data for the Indian population, which is essential for clinical and ergonomic assessment. Overall, the present study strengthens the importance of considering gender differences and bilateral assessment in the evaluation of lower limb biomechanics. The findings provide a foundation for future research and clinical applications aimed at improving knee health and preventing injuries.

Limitations 

The cross-sectional design confines the ability to establish causal relationships. The measurements were taken in a static position, which may not fully reflect dynamic knee biomechanics during functional activities. Additionally, factors such as physical activity level, muscle strength, and limb dominance were not extensively analyzed.

## Conclusions

The correlation between the Q-angle and ICD suggests that these parameters represent distinct aspects of knee alignment and independently contribute to knee biomechanics. These findings emphasize the importance of a comprehensive assessment of lower limb alignment, including both angular and linear parameters. The study provides valuable normative data for the adult Indian population and highlights the need for further research to explore dynamic and functional aspects of knee biomechanics.
